# COVID-19 and Age-Friendliness in Higher Education

**DOI:** 10.1093/geroni/igab046.2731

**Published:** 2021-12-17

**Authors:** Celeste Beaulieu, Nina Silverstein, Lauren Bowen, Susan Whitbourne, Joann Montepare

**Affiliations:** 1 University of Massachusetts Boston, Boston, Massachusetts, United States; 2 University of Massachusetts, Boston, University of Massachusetts Boston, Massachusetts, United States; 3 University of Massachusetts Boston, University of Massachusetts Boston, Massachusetts, United States; 4 Lasell University, Newton, Massachusetts, United States

## Abstract

During the COVID-19 pandemic, universities have changed to an online or hybrid format. These changes provide the opportunity for universities to be more accessible for all individuals. However, the logistics of university life during a pandemic has exposed significant and potentially enduring challenges and opportunities for designing and maintaining an Age-Friendly University. This study investigates perceptions of students, faculty, and staff in the lens of an age friendly university during the COVID-19 pandemic. This study draws on qualitative and quantitative data from over 10,000 faculty, staff, students, and life-long learners from 26 universities. Five items were asked to constituent groups about their perceptions on their university’s response to COVID-19. Overall, students had the poorest average perception of satisfaction with their university’s overall response to the pandemic, with 62% satisfied compared to 74% and 73% of faculty and staff, respectively. Further, 77% of faculty think the university’s response to continuing education accommodated their needs, compared to 69% of students. Respondents from private universities reported more positive satisfaction than respondents from public universities (M=4.23, SD=0.94; t(df=7405)=6.805, p<.001). Qualitative data suggest that older students and faculty needed more technological assistance during this transition to primarily online learning to keep older members involved in the community. Older staff felt that they were more likely to be furloughed and were the group most likely to not have a choice in working on or off campus.

